# Prenatal phenotype of PNKP-related microcephaly, seizures, and developmental delay: A case report and literature review

**DOI:** 10.1097/MD.0000000000041300

**Published:** 2025-01-17

**Authors:** Jin-Long Xie, Chun-Yan Jiang, Ping-Ping Sun, Yan Zhang, Na Sun, Su-Xian Luan

**Affiliations:** a The Reproductive Medicine Centre, Weifang People’s Hospital, Shandong Second Medical University, Weifang, Shandong, China; b Clinical Laboratory, Weifang People’s Hospital, Shandong Second Medical University, Weifang, Shandong, China.

**Keywords:** microcephaly, seizures, developmental delay, monogenic disease molecular genetic testing, PNKP gene, whole-exome sequencing

## Abstract

**Rationale:**

Microcephaly, epilepsy, and developmental delay (MCSZ) is a rare neurodevelopmental disorder associated with autosomal recessive inheritance of mutations in the polynucleotide kinase 3’-phosphatase (PNKP) gene. Prompt identification and management are essential, as delayed diagnosis or intervention may result in severe complications or mortality. In this case, prenatal screening in the second trimester detected fetal microcephaly with a gradual decline in head circumference, prompting the decision to terminate the pregnancy. Subsequent genetic analysis of the fetal tissue confirmed the presence of compound heterozygous mutations in the PNKP gene.

**Patient concerns:**

The patient, a 34-year-old remarried female with no history of consanguineous marriage, underwent 2 mid-trimester termination procedures due to fetal microcephaly and sought counseling for reproductive assistance.

**Diagnoses:**

The patient’s carrier status for PNKP mutations was ascertained through whole-exome sequencing of the termination tissue and molecular genetic testing for monogenic disorders. The terminated fetus was diagnosed with MCSZ, a condition associated with compound heterozygous mutations in the PNKP gene.

**Interventions:**

Fetal microcephaly was identified via mid-trimester prenatal ultrasound, leading to the termination of the pregnancy during the same trimester. Subsequent genetic analysis of the immediate family revealed compound heterozygous mutations in the PNKP gene as the underlying cause of MCSZ. Genetic counseling was provided, followed by 1 cycle of preimplantation genetic testing for monogenic.

**Outcomes:**

The patient carried the heterozygous c.1188 + 1G > A PNKP mutation, whereas her husband carried the heterozygous c.976G > A PNKP mutation. The fetus was found to have compound heterozygous mutations c.976G > A and c.1188 + 1G > A. After counseling, the couple underwent 1 cycle of preimplantation genetic testing for monogenic, unfortunately, no pregnancy occurred after the 2 embryos were transferred.

**Lessons:**

MCSZ, a condition caused by PNKP mutations, is exceedingly rare. Women with a history of adverse pregnancy outcomes should undergo close monitoring during prenatal checkups. If fetal microcephaly is detected, it is essential to strictly follow obstetric guidelines for prenatal care, such as comprehensive cranial magnetic resonance imaging and genetic testing for confirmation. Avoidance of consanguineous marriages is advised. Early detection and timely intervention are key to preventing adverse pregnancy outcomes.

## 1. Introduction

Microcephaly, seizures, and developmental delay (MCSZ, Online Mendelian Inheritance in Man #613402) is an autosomal recessive disorder associated with mutations in the polynucleotide kinase 3’-phosphatase (PNKP) gene.^[1,2]^ This rare neurodevelopmental condition presents with microcephaly, seizures, and varying degrees of psychomotor developmental delay, often resulting in severe complications or mortality. Previous literature indicated that prenatal fetal imaging abnormalities can manifest as parenchymal and cerebellar dysplasia, corpus callosum dysgenesis, ventriculomegaly, and abnormalities in skull development. Additionally, these abnormalities may present as microcephaly without associated brain development issues.^[3]^ Following birth, infants may display a variety of clinical phenotypes, including MCSZ, attention-deficit/hyperactivity disorder, hypotonia, gastroesophageal reflux, and hearing impairments.^[4]^ As previously reported, 3 missense variants of PNKP were also evaluated as likely pathogenic (c.526C > T, (p.L176F); c.968C > T, (p.T323M); c.976G > A, (p.E326K)), which could lead to corresponding clinical phenotypes.^[3]^ The mutation c.976G > A in PNKP identified in this case is a missense mutation and is among the most prevalent mutations associated with MCSZ. Prior studies have documented its correlation with delayed brain development, epilepsy, and microcephaly in postnatal infants, as well as early-onset infantile epileptic encephalopathy.^[5]^ In contrast, the variant c.1188 + 1G > A in PNKP is a splicing mutation that has not been previously reported in the literature regarding individuals affected by PNKP-related conditions. In this study, we present a case of mid-trimester pregnancy in which a fetus was diagnosed with microcephaly, leading to termination. Whole-exome sequencing of the termination specimen confirmed MCSZ due to compound heterozygous variants in the PNKP gene: c.976G > A (p.E326K) and c.1188 + 1G > A. To our knowledge, there have been no prior reports of fetal cases exhibiting compound heterozygous variants at these 2 loci. Therefore, the documentation of this case is crucial for enhancing the understanding of genetic disorders related to prenatal diagnosis and management, thereby contributing to the repository of genetic mutations.

## 2. Case presentation

### 2.1. Patient history

A 34-year-old female patient who had remarried was seeking to conceive again after undergoing termination of pregnancy twice in the middle trimester due to fetal microcephaly. She visited our hospital for genetic counseling and reproductive guidance on May 12, 2023. In 2019, following her remarriage, she conceived naturally, and prenatal screening at 5 months revealed fetal microcephaly, resulting in termination without prior chromosomal or genetic tests. In 2021, she again conceived naturally, and at 5 months of pregnancy, prenatal ultrasound revealed fetal microcephaly with progressive worsening. Unfortunately, the patient did not undergo fetal cranial magnetic resonance imaging (MRI). Another termination was performed, followed by whole-exome sequencing of the termination specimen and genetic testing for core family monogenic disease molecular genetic testing (Table 1). Notably, the patient has 1 healthy daughter from her previous marriage, and her male partner has 1 healthy daughter from his previous marriage.

**Table 1:** The timeline of different interventions for the patient.

Time	November 14, 2021 23 weeks of gestation	November 28, 2021 25 weeks of gestation	December 1, 2021	December 9, 2021	October 9, 2022	May 3, 2023	August 12, 2023
Interventions	Fetal systemic ultrasound	Prenatal ultrasound	Genetic counselling	Termination of pregnancy and whole-exome sequencing	Molecular genetic testing for single-gene disorders	Pre-pregnancy genetic counselling	PGT-M

PGT-M = preimplantation genetic testing for monogenic.

### 2.2. Prenatal ultrasound

**Figure 1.**
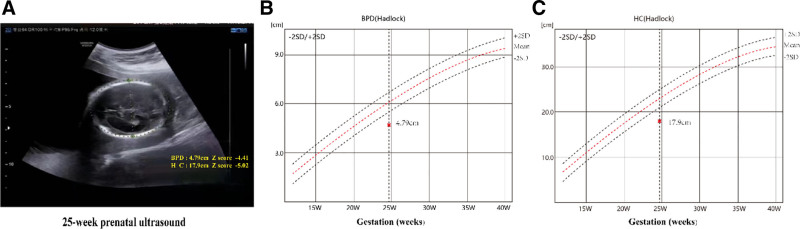
Prenatal ultrasound image and growth curve of the fetus at 25 weeks of gestation. The chart depicts the growth trajectories of head circumference and BPD throughout pregnancy including the standard growth curve along with plus and minus 2 SDs, which serve as a means to identify potential abnormalities in fetal growth and development. (A) The prenatal ultrasound at 25 weeks revealed a BPD of 4.79 cm and an HC of 17.9 cm. (B) The growth curve for BPD showed that at 25 weeks of gestation, the fetal BPD was 4.41 SDs below average. (C) The growth curve for HC indicated that at 25 weeks of gestation, the fetal HC was 5.02 SDs below average. BPD = biparietal diameter, HC = head circumference, SDs = standard deviations.

Multiple prenatal ultrasound follow-up visits during pregnancy consistently indicated that the fetal head was underdeveloped, as evidenced by measurements such as head circumference falling below normal ranges for gestational age. The head circumference exhibited slow growth on the growth curve, with the biparietal diameter (BPD) measuring 4.41 standard deviations below average by 25 weeks of gestation, the head circumference was 5.02 standard deviations below average, and the abdominal circumference measured 17.5 cm, which was 2.33 standard deviations below average. In contrast, the fetal femur length was 4.3 cm, falling within the normal range (Fig. 1).

### 2.3. Whole-exome sequencing and core family monogenic disease molecular genetic testing were conducted on fetal tissue

**Figure 2.**
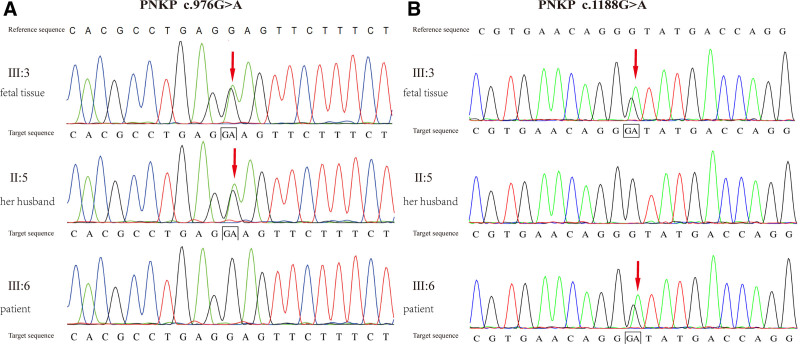
Whole-exome sequencing results. (A) The fetus and the patient’s husband carried the PNKP c.976G > A mutation (II:5, III:3). (B) The fetus and the patient carried the PNKP c.1188G > A mutation (II:6, III:3). PNKP = polynucleotide kinase 3’-phosphatase.

According to the reference genome GRCh37/hg19, whole-exome sequencing of fetal tissue identified compound heterozygous mutations in the PNKP gene: c.976G > A (p.E326K) and c.1188 + 1G > A. The patient carried the PNKP c.1188 + 1G > A mutation, and her current husband carried the PNKP c.976G > A (p.E326K) mutation (Fig. 2). Both mutations are pathogenic variants associated with MCSZ. The c.976G > A mutation is a missense mutation, causing an alteration in the amino acid sequence. In contrast, the c.1188 + 1G > A mutation affects the splice site, potentially resulting in abnormal mRNA splicing. Functional studies have demonstrated that the c.976G > A (p.E326K) variant in the PNKP gene impairs protein function (PS3). This variant is not included in the GnomAD database, indicating its rarity in the general population (PM2_P). Literature reports have identified this variant as homozygous in individuals with microcephaly, seizures, and developmental delay (PM3), and it has been found to segregate with the disease within families (PP1_Supporting). According to the ACMG guidelines, this variant is classified as pathogenic (PS3 + PM2_P + PM3 + PP1_S).

**Figure 3.**
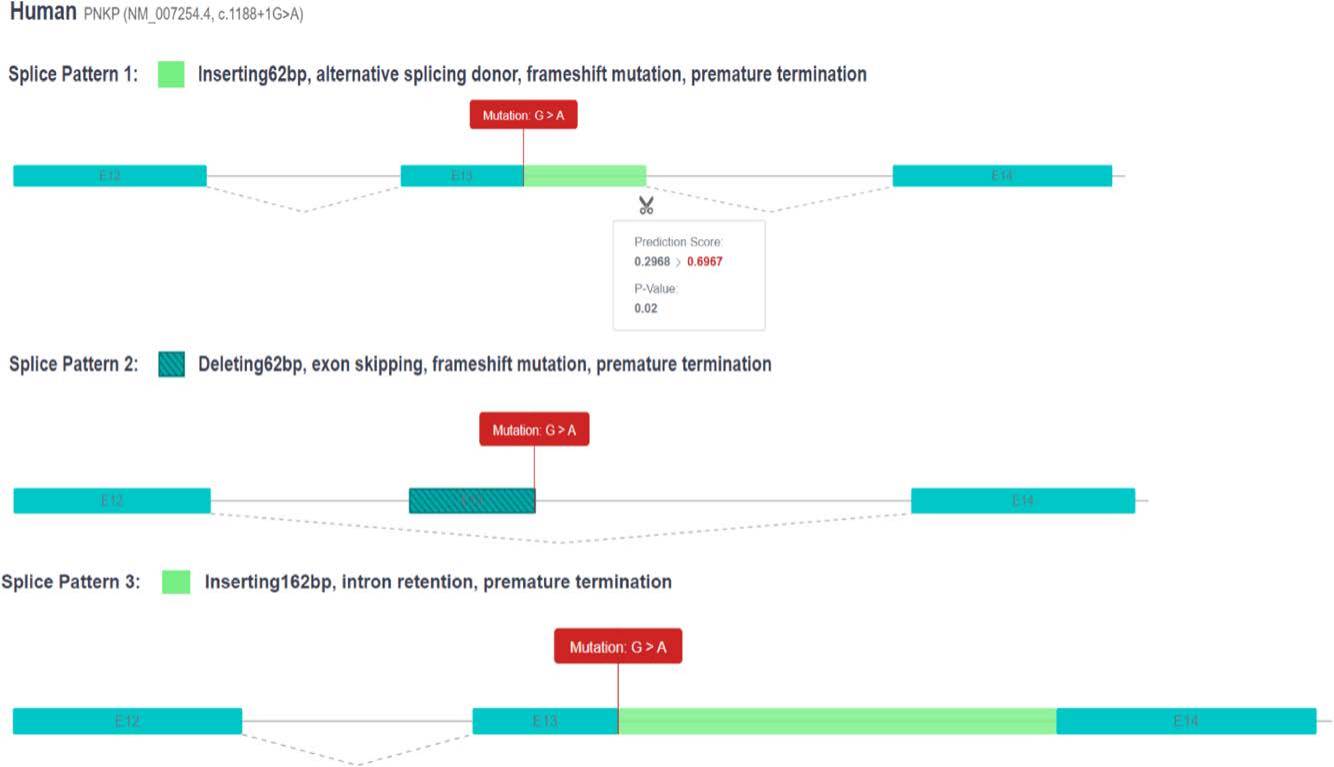
Three splice patterns of PNKP (NM_007254.4, c.1188 + 1G > A). PNKP = polynucleotide kinase 3’-phosphatase.

The PNKP c.1188 + 1G > A variant (NM_007254.4) is a splice-site mutation that likely leads to abnormal gene splicing, thereby affecting gene function (PVS1). This variant is associated with 3 splice patterns: (1) the insertion of 62 bp leading to alternative splicing donors, frameshift mutation, and premature termination; (2) the deletion of 62 bp resulting in exon skipping, frameshift mutation, and premature termination; and (3) the insertion of 162 bp causing intron retention and premature termination (Fig. 3). Similar to the c.976G > A variant, this mutation is also not reported in the GnomAD database, indicating its rarity in the general population (PM2_P). In tissue from induced abortions, a pathogenic variant was identified in trans with this variant (PM3). In accordance with the ACMG guidelines, this variant is classified as pathogenic (PVS1 + PM2_P + PM3).

**Figure 4.**
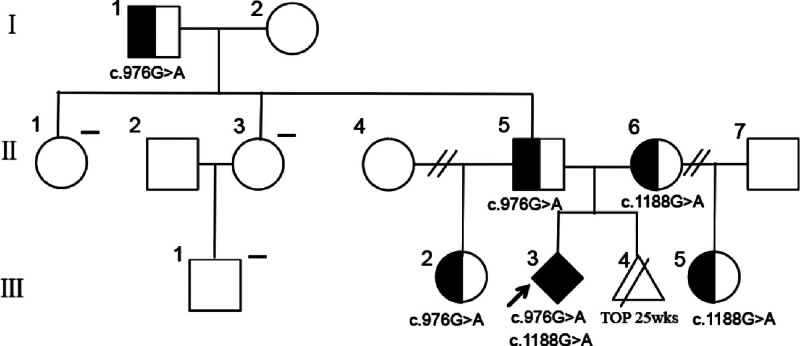
Core pedigree chart. The fetus had compound heterozygous PNKP mutations c.976G > A (p.E326K) and c.1188 + 1G > A (III:3). The patient and her daughter from a previous marriage carried the PNKP c.1188 + 1G > A mutation (II:6, III:5). TOP 25wks: Termination of pregnancy at 25 weeks (III:4). The patient’s current husband, his father, and his daughter from a previous marriage carried the PNKP c.976G > A mutation (I:1, II:5, III:2). (‐): Negative for PNKP gene mutation (II:1, II:3, III:1). E = glutamic acid, K = lysine, PNKP = polynucleotide kinase 3’-phosphatase.

**Figure 5.**
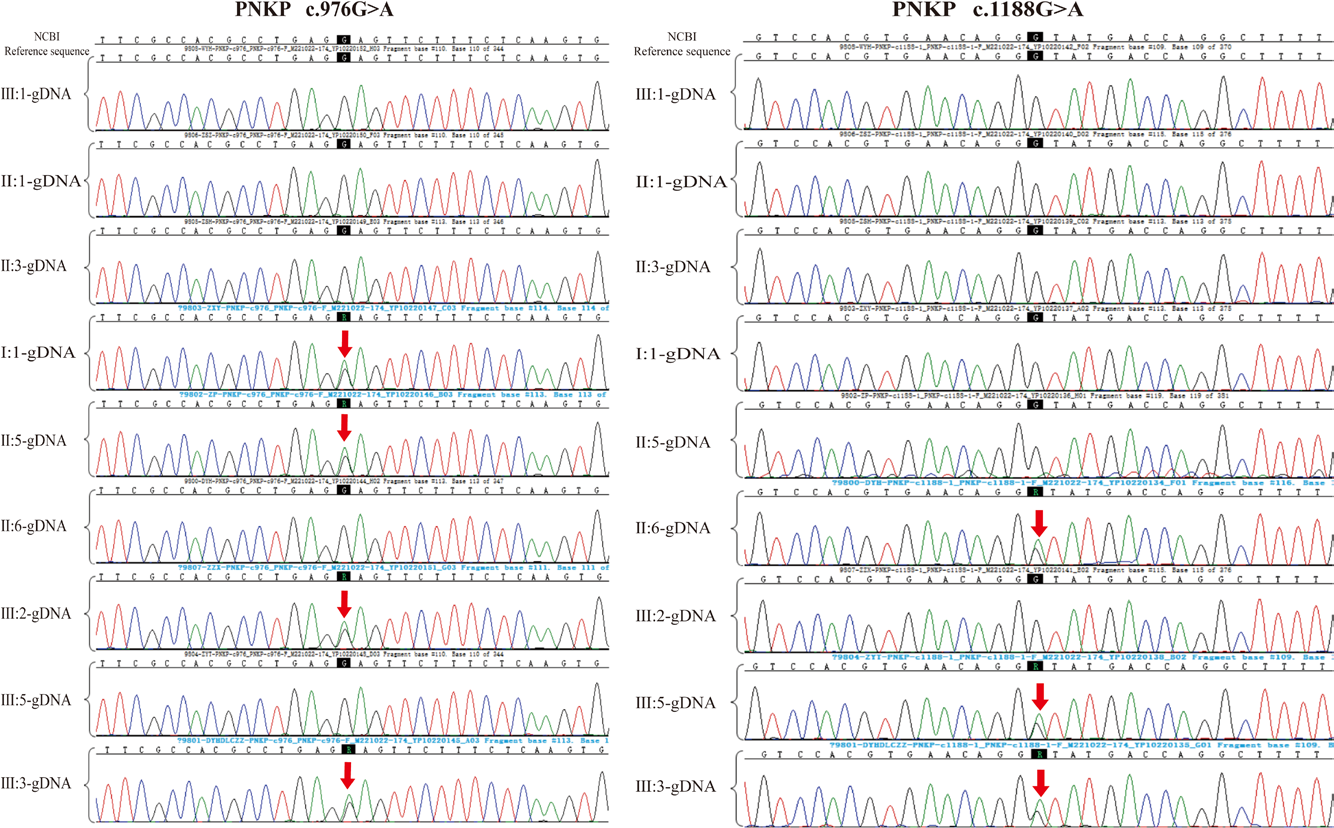
Results of molecular genetic testing for monogenic disease: PNKP c.976G > A (p.E326K) and PNKP c.1188 + 1G > A. E = glutamic acid, K = lysine, PNKP = polynucleotide kinase 3’-phosphatase.

Monogenic disease molecular genetic testing identified a heterozygous c.1188 + 1G > A mutation in the PNKP gene in the patient. This mutation was also found in the patient’s daughter from a previous marriage. Additionally, the patient’s current husband was found to carry a heterozygous c.976G > A mutation in the PNKP gene, which was also present in his father and daughter from a previous marriage. Despite carrying these mutations, not all individuals exhibited clinical symptoms. Notably, neither the husband’s 2 sisters nor his nephew carried the PNKP gene mutation (Figs. 4 and 5).

### 2.4. AlphaFold2.3.0 software was used to predict 3D protein structures at the mutation sites

**Figure 6.**
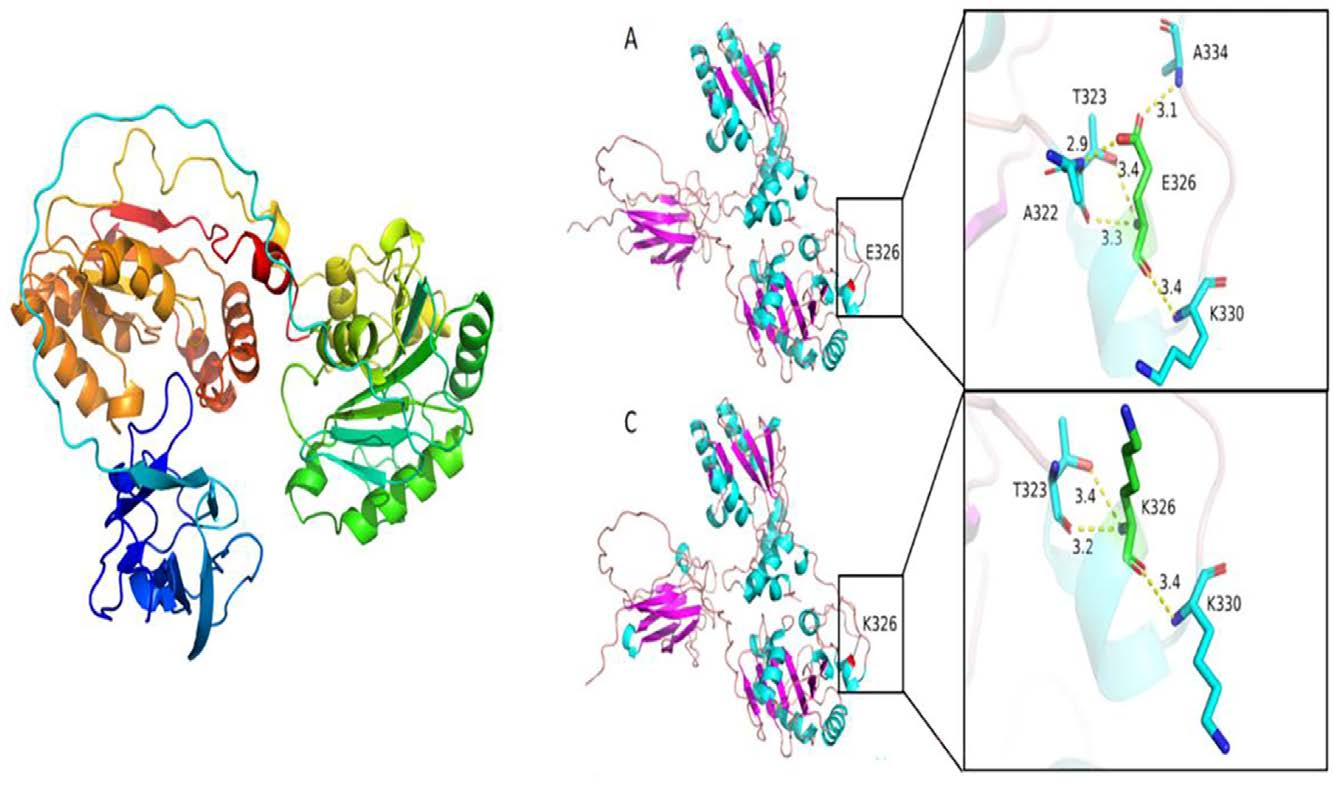
3D protein modeling of PNKP missense variants p.E326K. The E326K protein mutation causes changes in charge and interactions, leading to alterations in protein function. Protein 3D molecular modeling was predicted by AlphaFold2.3.0 software. E = glutamic acid, K = lysine, PNKP = polynucleotide kinase 3’-phosphatase.

With the PNKP E326K mutation, notable alterations in interactions were observed. In the wild-type protein, residue 326 was occupied by the negatively charged polar amino acid glutamic acid. E326 establishes hydrogen bonds with K330, A334, T323, and A322 at bonding distances of 3.4, 3.1, 3.4, 3.3, and 2.9 Å, respectively. The E326K mutation substitutes glutamic acid with lysine. The mutated K326 site forms hydrogen bonds with K330 and T323, with bond distances of 3.4, 3.4, and 3.2 Å. This mutation modifies the charge properties and interactions of the side chain, ultimately resulting in functional changes (Fig. 6).

### 2.5. Follow-up

The patient underwent 1 cycle of preimplantation genetic testing for monogenic (PGT-M), assisted reproduction treatment at an external hospital. A total of 4 oocytes were retrieved, leading to the generation of 2 embryos. Unfortunately, no pregnancy was achieved after embryo transfer. The patient and her husband subsequently chose to cease any additional assisted reproductive treatments.

## 3. Discussion

Fetal microcephaly is defined as a significantly smaller head circumference of 3 SDs below the median for a specific age, sex, and race compared with other fetuses at the same gestational age. This deviation signals abnormal brain development and is linked to various neurological outcomes, such as epilepsy, cerebral palsy, and intellectual disabilities. Rather than a standalone diagnosis, it is considered a clinical symptom that often results from factors affecting brain development. A cohort study of 680 cases revealed that genetic factors accounted for approximately 50% of cases, whereas perinatal brain injury, including infections and teratogenic exposures, accounted for approximately 48%.^[6]^ Specific gene mutations, such as PNKP, PIK3R1,^[7]^ PPP5C,^[8]^ INPP4A,^[9]^ HMGB1,^[10]^ NUSAP1,^[11]^ and CHKA,^[12]^ are associated with this condition. Nongenetic causes include infectious agents such as cytomegalovirus,^[13]^ toxoplasmosis, rubella, herpes, syphilis, human immunodeficiency virus, and Zika virus^[14]^; maternal exposure to substances such as heavy metals, alcohol, and smoking during pregnancy; and perinatal brain injuries such as hypoxia, ischemia, or trauma.

To improve diagnostic accuracy, a comprehensive assessment is recommended when fetal microcephaly is suspected clinically.^[15]^ This assessment includes verifying gestational age using the early pregnancy crown–rump length or fetal biometric measurements and inquiring about the pregnant woman’s medical and surgical history and exposure to teratogenic substances. Family history within 3 generations should be considered, and the head circumference of the fetus’s parents should be measured. Prenatal ultrasound and prenatal brain MRI, which focus on brain imaging features such as cerebrovascular abnormalities, ventricular enlargement, calcification, and hemorrhage,^[16]^ are crucial procedures during checkups. TORCH screening, especially for congenital cytomegalovirus infection, and Zika virus testing should also be performed depending on exposure history and clinical symptoms.^[17]^ If ultrasound reveals a fetal head circumference <4 SDs, or <3 SDs with the presence of other abnormalities, fetal MRI should be conducted to confirm any additional brain abnormalities. Ideally, fetal MRI should be performed between 28 to 32 weeks of gestation to obtain detailed information on fetal brain anatomy. Fetal microcephaly necessitates evaluation by a medical geneticist or through autopsy, including systematic chromosome testing, rapid aneuploidy detection/microarray, or molecular studies for single-gene diseases related to neuronal migration disorders or cortical malformations.^[18–21]^ For patients opting to continue pregnancy, regular follow-up is vital to monitor the fetal head growth curve, brain disease development, and overall fetal health.

Whole-exome sequencing and molecular genetic testing identified compound heterozygous mutations in the PNKP gene as the cause of the patient’s condition, leading to MCSZ. MCSZ, a rare genetic disorder initially described in 2010, arises from variations in the PNKP gene located on chromosome 19q13.33.^[2]^ This gene, spanning 8.3 kb with 17 exons, encodes a 521 amino acid protein. The protein contains forkhead-associated (FHA), kinase, and phosphatase domains, which are crucial for the interactions of PNKP with other DNA repair proteins involved in single-strand break repair and nonhomologous end joining repair.^[22,23]^ Dysfunction in DNA repair mechanisms can impact neuronal function, as neurons, which are highly transcriptionally active and energy-demanding cells, generate reactive oxygen species during metabolism.^[24]^ The accumulation of unrepaired damaged DNA can directly affect neuronal function. Studies have associated the loss of PNKP enzyme activity with immunodeficiency and tumor susceptibility,^[25]^ including high-grade brain tumors associated with PNKP gene mutations.^[26]^

Microcephaly may arise from an inadequate production of neurons during development or from degeneration following normal development. This suggests that microcephaly can result from the apoptosis of precursor cells, differentiated neurons, or both cell types. Mutations associated with MCSZ lead to a reduction in the cellular stability and levels of the PNKP protein.^[2]^ To date, 4 mutations in PNKP have been identified in cases of MCSZ. The L176F and E326K mutations are point mutations occurring at highly conserved residues within the DNA phosphatase domain.^[27]^ Mutations in the PNKP gene, which encodes a critical enzyme involved in DNA repair, are associated with several neurodegenerative disorders, including MCSZ.^[1,3,28]^ The PNKP enzyme exhibits essential 3’-phosphatase and 5’-kinase activities necessary for repairing both single-strand and double-strand DNA breaks.^[27,29]^ Mutations such as c.976G > A, resulting in an arginine-to-glutamine substitution, can alter the enzyme’s structure, while splice-site mutations like c.1188 + 1G > A can disrupt mRNA splicing, leading to reduced levels of functional protein. These mutations primarily result in decreased PNKP activity, thereby impeding DNA damage repair, which is critical for neuronal proliferation and function. The absence of effective DNA repair mechanisms leads to the accumulation of DNA damage, cell cycle abnormalities, apoptosis, and neuronal dysfunction. Among the clinical manifestations, severe seizures distinctly highlight the consequences of PNKP mutations in contrast to other DNA repair deficiencies. Moreover, data suggest that impaired single-strand break repair (SSBR), which may convert single-strand breaks into double-strand breaks during DNA replication,^[30]^ could be a critical factor in the neurodevelopmental and neurodegenerative pathologies observed in PNKP-associated disorders.^[31]^ Consequently, the reduction in SSBR, rather than defects in double-strand break repair, appears to be the underlying cause of these conditions, with severity determined by the extent and nature of the reduction in repair capacity. Missense variants, such as T323M, likely decrease PNKP levels by affecting protein stability, further supporting the notion that compromised SSBR contributes to the disease pathology.^[26]^

Compound heterozygous mutations in the PNKP gene impact DNA repair, resulting in neurodevelopmental defects and various clinical presentations, such as MCSZ,^[2]^ ataxia with oculomotor apraxia type 4,^[32–34]^ and Charcot–Marie–Tooth disease type 2B2.^[35]^ Therefore, a comprehensive assessment of pathogenic factors should consider the patient’s clinical symptoms, disease-causing genes, and relevant biochemical markers. In this patient, genetic analysis revealed that the c.976G > A and c.1188 + 1G > A mutations in the PNKP gene were inherited from the father and mother, respectively, in a heterozygous state, indicating autosomal recessive inheritance of MCSZ in the family. Clinical manifestations occur with compound heterozygosity, where carriers of 2 different mutated alleles exhibit symptoms. Genetic testing is crucial for suspected cases, with genetic counseling recommended for parents during future pregnancies to reduce the risk of MCSZ and enhance the quality of life for future offspring.

Upon the identification of microcephaly, amniocentesis is advised for prenatal chromosomal analysis and whole-exome sequencing to identify potential causative genes and associated disorders. Additionally, a cranial MRI may be conducted to evaluate cortical development and identify abnormalities such as ventriculomegaly. Establishing a definitive diagnosis facilitates timely genetic counseling, and the option of pregnancy termination may be considered if deemed necessary. Families with PNKP gene mutations should receive comprehensive genetic counseling to evaluate the risk of recurrence. Preconception and prenatal whole-exome sequencing, along with noninvasive prenatal testing can assist in identifying carriers and offer reproductive options for couples at high risk, including in vitro fertilization and PGT, thereby enhancing the likelihood of having a healthy offspring. The development of artificial intelligence (AI) technologies, particularly advancements in machine learning algorithms, has enabled scientists to decipher complex patterns in the genome and design synthetic DNA sequences that can precisely regulate gene expression in specific cell types.^[36]^ Researchers, including Gosai, have employed AI-assisted design to achieve enhanced control of gene expression in targeted cell types, which is anticipated to minimize off-target effects in gene therapy, thereby improving the safety and efficacy of treatments.^[37]^ The synthetic sequences generated by AI exhibit greater specificity in driving gene expression in target cells compared to any regulatory elements found in nature. This advancement also provides a theoretical foundation for future gene therapy applications in MCSZ. Furthermore, individuals diagnosed with MCSZ should undergo thorough neurodevelopmental evaluations and receive supportive treatments, which may encompass epilepsy management, physical therapy, speech therapy, and educational assistance.

## 4. Conclusions

For families with a history of microcephaly, particularly those with identified mutations in genes such as PNKP, it is essential to compile comprehensive medical histories. This compilation should include inquiries regarding consanguineous marriages and hereditary diseases. Additionally, it is important to conduct screenings for blood pressure, blood sugar, weight, and thyroid function, as well as to evaluate folate metabolism. Women are recommended to take an appropriate dosage of folic acid 3 months prior to conception to prevent neural tube defects, mitigate birth defects, and enhance overall population health. Families with PNKP gene mutations should receive comprehensive genetic counseling to evaluate the risk of recurrence. Preconception and prenatal whole-exome sequencing can assist in identifying carriers and offer reproductive options for couples at high risk, such as in vitro fertilization and PGT, thereby increasing the likelihood of having a healthy offspring. Furthermore, individuals diagnosed with MCSZ should undergo comprehensive neurodevelopmental evaluations and receive supportive treatments, which may encompass epilepsy management, physical therapy, speech therapy, and educational assistance.

The occurrence of MCSZ caused by compound heterozygous mutations in the PNKP gene is remarkably rare. This report highlights a case of MCSZ resulting from compound heterozygous mutations in the PNKP gene, emphasizing the critical function of PNKP in DNA repair and neurodevelopment. By conducting trio whole-exome sequencing, the hereditary pattern of this disorder was clarified, enabling the proposal of clinical management and prevention strategies. Comprehension of the diverse pathogenicity and anticipated symptoms is crucial for improving counseling during the prenatal stage, navigating the postnatal period, facilitating prenatal diagnosis in future pregnancies, and potentially opening avenues for future treatments.

From the patient’s perspective:

The patient expressed that for any future pregnancies, they would not proceed without prior consultation and examination at a medical facility. They intend to adhere to the doctor’s recommendations, undergo appropriate pre-pregnancy screenings, and, if necessary, seek genetic counseling. Based on the genetic counselor’s guidance, they would contemplate undergoing prenatal chromosomal and genetic testing, along with related genetic evaluations. Additionally, they aim to select suitable assisted reproduction strategies to mitigate adverse pregnancy outcomes and minimize the risk of birth defects.

## Acknowledgments

We thank the patient and his family for their participation in this study. We thank Professor Yu-Jie Zhang and Zhen-Sen Gao from the Department of Ultrasound of Weifang People’s Hospital for the guidance and assistance with this article.

## Author contributions

**Conceptualization:** Jin-Long Xie, Su-Xian Luan.

**Data curation:** Jin-Long Xie, Ping-Ping Sun, Yan Zhang, Na Sun.

**Formal analysis:** Jin-Long Xie.

**Investigation:** Jin-Long Xie, Chun-Yan Jiang.

**Software:** Jin-Long Xie, Chun-Yan Jiang.

**Supervision:** Su-Xian Luan.

**Validation:** Jin-Long Xie, Chun-Yan Jiang, Ping-Ping Sun, Yan Zhang, Na Sun, Su-Xian Luan.

**Writing – original draft:** Jin-Long Xie.

**Writing – review & editing:** Jin-Long Xie, Chun-Yan Jiang, Ping-Ping Sun, Yan Zhang, Na Sun, Su-Xian Luan.
